# Experiences with use of technology and telehealth among women with perinatal depression

**DOI:** 10.1186/s12884-022-04889-4

**Published:** 2022-07-18

**Authors:** Uma D. Parameswaran, Ryoko Pentecost, Marcia Williams, Marcela Smid, Gwen Latendresse

**Affiliations:** 1 Department of Psychology, University of Utah, Salt Lake City, USA; 2 Department of Applied Psychology, New York University, New York, USA; 3 College of Nursing, University of Utah, Salt Lake City, USA; 4 Maternal Fetal Medicine, University of Utah Hospital, Salt Lake City, USA

**Keywords:** Telehealth, Perinatal depression, Depression, Videoconference

## Abstract

**Background:**

Perinatal depression (PD) affects 10–20% of childbearing women. Telehealth is increasingly utilized for mental health services to increase access to care and overcome COVID-19 pandemic barriers. Women’s perspectives on telehealth services for PD is unknown, however. This study’s primary objective was to obtain the perspectives of women who participated in an 8-week group videoconference intervention for PD symptoms, including how technology impacted their experience.

**Methods:**

We utilized theoretical sampling and included perinatal women who had completed the 8-week mindfulness-based cognitive-behavioral intervention group. Semi-structured focus groups with four to six women were conducted on a videoconference platform. Primary analysis used grounded theory and a secondary analysis used qualitative description and was conducted by two coding teams. The teams collaborated on the final themes across the analyses.

**Results:**

Three groups, with a total of 17 participants were conducted. Composition consisted of seven postpartum and ten pregnant women from the 47 total participants. Identified core themes regarding their experiences of the videoconference intervention were: positive experiences, negative experiences, suggestions and ideas, and screening and communication.

**Conclusion:**

This study provides growing evidence informed by perinatal women of positive experiences with engagement in a videoconference intervention for PD. Telehealth may be a reasonable and acceptable platform to increase access and retention for mental health services in childbearing women. Further, this pilot work showcases videoconferencing delivery for a wide range of effective and affordable mental health services in low-resource communities.

## Introduction

Perinatal depression (PD) affects 10–20% of childbearing women [1, 2]. Approximately 40% of women with family history of depression, major life events, or from disadvantaged socioeconomic groups experience PD [2–6]. PD is associated with adverse outcomes, such as low birthweight, preterm delivery, breastfeeding cessation, and higher rates of maternal smoking and maternal suicidal ideation and suicide [7–11]. The United States Preventative Services Taskforce recommends universal screening for PD for all pregnant and postpartum women, but has not addressed the issue of access to mental health resources for women who screen positive [12–14]. There is a shortage of mental health providers, particularly in low-resource and rural settings, and within minority populations [15, 16]. An additional barrier is the lack of providers with knowledge specific to PD [17–19].

Telehealth is revolutionizing the delivery of healthcare services, and is a promising platform for mental healthcare [20]. Telehealth has rapidly become an essential tool for delivering care during the COVID-19 pandemic [21]. Telehealth connects patients and providers remotely using broadband access, regardless of geographical location of individuals. Much of telehealth has focused on individual service delivery rather than utilizing a group format [22].

Limited research exists related to feasibility and acceptability of telehealth among childbearing women. In 2012 the Institute of Medicine [23] addressed the need to evaluate the effectiveness of telehealth and urged researchers and funding bodies to follow the recommendations [24] to include “the use of more naturalistic methods and settings,” and “better standardization of populations, interventions, and outcome measures.” To address this issue, we evaluated a group videoconference intervention (VCI) to deliver mental health services to women with mild to moderate PD symptoms [25]. The purpose of our subsequent qualitative study was to obtain the perspectives of the women who participated in these groups. The aims of the study were to: 1) describe the perspectives of women who participated in the VCI; 2) describe the women’s recommendations for how to improve the intervention, and 3) conduct a secondary data analysis specifically focused on the impact of use of technology. The study received approval from the University of Utah Institutional Review Board (IRB_00071041).

## Methods

### Videoconference intervention/UPLIFT program

Women who participated in the eight-week VCI were either pregnant or postpartum and had screened positive for mild to moderate depression using Edinburgh Postnatal Depression Scale (EPDS) screening tool [26] or were at high risk for developing depression. A full description and results of that study are published [27]. Briefly, intervention groups (four to six women) met for 1 h for 8 weeks by secure videoconference, managed by the Utah Telehealth Network [28]. The groups were facilitated by a mental health professional using a manualized program based on mindfulness-based practices and cognitive behavioral therapy (MBCBT) [29, 30]. Each session included topics such as thought monitoring, identifying cognitive distortions, goal setting and mindfulness exercises (e.g. body scan, progressive muscle relaxation, and meditation).

After completing the 8-week VCI, women were invited to participate in a focus group to discuss their perspectives about the intervention. The research team conducted semi-structured focus groups (three) via videoconference to receive input directly from women who had participated in the eight-week VCI. Each focus group was facilitated by a PhD-prepared psychologist-researcher. The questions for the focus-group guide were based on the feedback we received from the facilitators who conducted the intervention. The focus groups were recorded, accompanied by notes from the focus group facilitator, and transcribed. The focus group interview schedule (Table 1) included questions about women’s experiences with the virtual intervention, barriers to participation, and recommendations for intervention improvement.

**Table 1:** Focus group interview schedule

Items	
What worked well for you?	
What didn’t work well for you?	
What are your thoughts about screening?	
Tell us about the interaction with your doctor or midwife	
Tell us your thoughts about the group facilitators	
Content: what did you like most?	
Content: what did you like least?	
Between sessions: how did it go?	
Between sessions: Which practices did you do?	
Between sessions: Favorites?	
Between sessions: Least favorites?	
Participant Manual: what did you think? Was it helpful? Not helpful?	
If you were not able to attend some (or any) of the meetings, please tell us what prevented you from attending?	
What are your suggestions for improvements?	
Any additional comments?	

### Primary qualitattive data analysis

Three qualitative researchers completed data analysis using a grounded theory approach to analyze data collected through semi-structured focus groups. An inductive approach by Corbin and Strauss [31] was used to analyze the data. The research team used a comparative consensus method after open coding to develop axial and selective codes. After transcription, the qualitative research team identified significant content areas and emerging themes [31]. First, each team member individually coded using open coding, followed by team agreement on the emerging themes. Subsequent discussion led to additional codes, collapsing of codes, or rewording the codes used in further analysis of the transcripts, and leading to themes. Important themes obtained from earlier focus groups were introduced in subsequent focus groups to obtain iterative feedback [31]. Once data collection was completed, all data were analyzed as a whole, for major content areas, core themes, and a central theme.

### Secondary data analysis

A secondary analysis of the focus group data was conducted using a qualitative description method in order to *describe* women’s experiences rather than assess and quantify those experiences. The method is a useful for guiding the development/adaption of future interventions targeting the same population. In this secondary analysis, we used transcriptions and subjects to perform inductive content analysis, constant comparison, and coding using Dedoose qualitative software. Although, the qualitative process was similar to the primary analysis, the focus of the secondary analysis was aimed at understanding the use of technology in enhancing (or diminishing) community and connection. The research team used the Consolidated Criteria for Reporting Qualitative Research checklist [28] for reporting study methods and results.

## Results

Of the 47 women who participated in the VCI, 17 (36%) agreed to participate in a focus group. Seven women were postpartum (1–5 months) and ten were pregnant (average gestational age, 22.7 weeks; SD, 10.6; range, 3–40 weeks). The mean age of participants was 30.9 years, with a range of 23 to 38, and all 17 reported being married or living with a partner. The EPDS scores ranged from 2 to 23 pre-intervention, and 1–17 post-intervention. For the 47 women, post-intervention PD symptoms decreased for those with mild to moderate symptoms of depression while those who were asymptomatic, but with high-risk factors for developing depression, remained largely asymptomatic [25]. Characteristics of the participating women are provided in Table 2.

**Table 2:** Focus group participant characteristics

***Characteristics***	***Number***
Ethnic/Racial Background	
White, Not Hispanic	15
Hispanic	1
African American	1
Annual Income	
$10–20,000	1
$40–50,000	2
$50–60,000	1
$60–70,000	3
$70–80,000	1
$80–90,000	1
$100,000	8
Education	
Not reported	2
Some College	1
College Graduate	7
Post Graduate Master’s Degree	5
Doctorate Degree	2

**Fig. 1:**
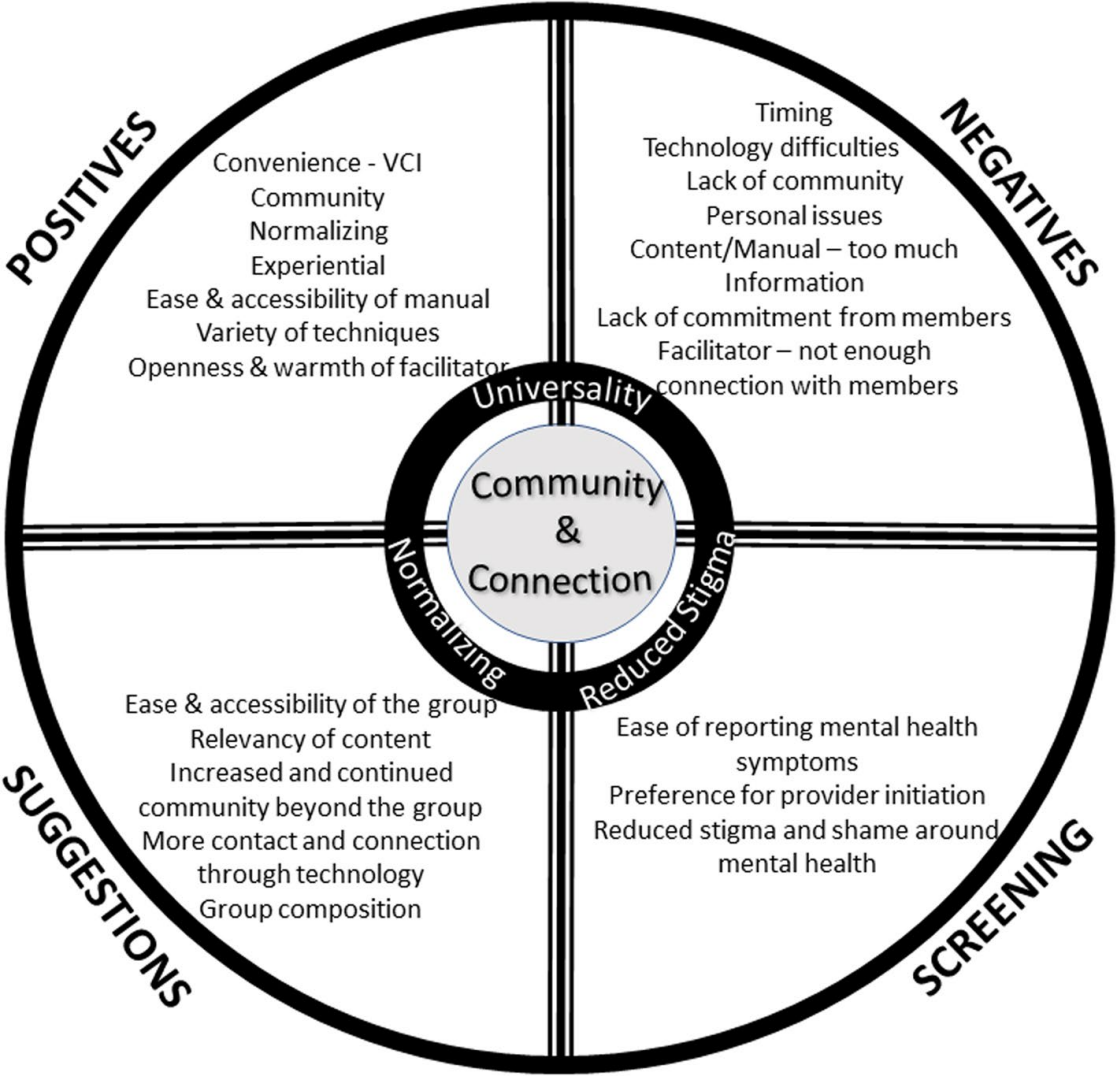
Primary analysis thematic diagram

After multiple iterations by the coding team, the data were organized into four major content areas after the primary analysis using grounded theory: (1) positive experiences, (2) negative experiences; (3) suggestions and ideas; and (4) screening and communication. As a result, three core themes emerged (Universality; Normalizing; and Reduced Stigma) across the four content areas with one central theme (Community & Connection) encompassing the three core themes (Fig. 1). Examples of the participant’s comments are included in tables for each content area. Screening and communication themes were centered on participant’s experiences to providers and how they screened for PD which was not directly related to the intervention. For the purposes of this paper, we are focusing specifically on the themes related to the use of technology .

### Content areas

#### Positive experiences

Important themes reflecting positive experiences emerged from the focus groups: convenience of attending sessions via VC, community building, normalization of feelings, and learning a variety of techniques for reducing PD symptoms. Each participant provided at least one positive comment relating to her participation in the VCI (Table 3).

**Table 3:** Positive experiences

	Participant Quotes
Community	*“I really enjoyed the group setting. It was nice to be able to connect with other people who were going through similar things.”*
Normalizing	*“I really like that safe space with people who are going through similar things, to me, and a place where I really could just be open and honest about what I was feeling about.”*
Variety of Techniques	*“It was interesting in our group like everybody liked different* *Things which was great that there were so many to try…I think because you could just experiment with them and see different things that work, I like that.”*

#### Negative experiences

Participants offered insight on negative experience**s** during the eight-week intervention: technological barriers, inability to connect with women outside of the VCI groups, lack of commitment from other group members, feeling overwhelmed by the number of techniques introduced, timing/scheduling of focus group sessions, lack of personal motivation, and perception of lack of time to adequately engage with the facilitator (Table 4).

**Table 4:** Negative experiences

	Participant Quotes
Use of Technology	*“The technology… I try to be 20 minutes early for this and I was 10 minutes late, just finding the email, getting in, getting the passwords to work. It’s usually annoying and it was occasionally prohibitive.”*
Lack of Community	*“I had hopes that there would be more interaction with the group and not just the singular like the instructor structure and the instruction.”*
Content/Manual: Too Much Information	*“…there were so many different methods that I didn’t feel like* *I got to try them all out.”*
Facilitator: Not Enough Connection with Members	*“I think we ran out of time a lot and maybe it’s just figuring out the material or figuring out how much conversation needs to be had or maybe there’s just the opportunity to talk afterward. I mean, she was open if we needed to talk but I think for us, it was kind of time prohibitive.”*

#### Areas for improvement

Participants identified several areas for improvement: recording of sessions for later viewing when unable to join a particular session, follow-up sessions with structured assignments, an option for connection with one another outside of group sessions, and offering different groups to address specific mental health needs, such as depression or anxiety (Table 5).

**Table 5:** Suggestions for improvement

	Participant Quotes
More Contact and Connection through Technology	*“I don’t know if it’s allowed because it’s a study but maybe if there was some sort of maybe a private Facebook group or something where you could connect to the people throughout the week in between meetings just so if you want a little bit of extra peer support you could reach out to them or just share a bad day with them if they wanted to respond they could.”*
Group Composition	*“I would say, anything to make it more specialized. I know some people in the group struggled more with depression, some were with anxiety, maybe getting all the people that mainly dealt with depression, people that mainly dealt with anxiety just because there are so many different situations and not enough time so just making it more specialized that way.”*

##### Core and central themes

In each of the four major content areas, three core themes emerged including universal need for community among women with PD, normalizing feelings of depression, and reducing the stigma of depression. The VCI provided a way to normalize depression, and made participants feel less isolated, knowing that there were others with similar experiences. The emerging central themes amongst the four areas focused on women feeling a connection to each other and the facilitator. This connection was either enhanced or diminished based on their experience of the use of technology.

##### Results from secondary data analysis

A total of six categories were identified during the secondary analysis and these were similar to the results of the primary analysis; use of technology, community, keeping connection, program content, program organization, and learning style. However, two of the six categories, community and keeping connection, were found to have significant interactions with the category of use of technology. Thus the results of the secondary analysis focus on describing how “use of technology” positively and negatively impacted community and connection between participants.

Participants reported convenience of using technology (i.e. use of their own device and attending via videoconference) in group sessions, which included the convenience of being in a private setting rather than a provider’s office (exemplar codes: private setting), not needing to leave the home setting, ability to join the group from any remote location using an internet connection (don’t need to leave home), and easier accessibility and flexibility in the video chat system (accessibility).

“*Technology is what makes this program great and the fact that we could do it from home like that, flexibility was huge for me.”*

*“The only reason I said yes is because I could go to my master bedroom, lock the door and I didn't have to find a babysitter, I didn't have to schedule an appointment and coordinate like… to get somewhere out of the house. It was just really nice; I could nurse my baby and be in group.”*

Although some participants expressed positive feedback relating to the use of technology, other participants experienced frustration when using the technology. Some participants described difficulties logging into the web-based video chat or experienced an audio or video malfunction., These incidents led to negative technological experiences in the larger groups as a whole. Due to technological issues, for example, multiple group sessions resulted in a later start time than normal (delayed start). Participants also expressed that the facilitator sometimes experienced technological difficulties requiring them to cancel classes, but notifications were not broadcasted to other group members (facilitator miscommunication). Moreover, participants described their frustration with technological challenges as “perfect storms” and said, “technology was a drag.” These expressions showcased the stress and barriers participants experience with use of technology.

*“The [use of] technology. I mean, I know it's part of it but ... Even just now, I try to be 20 minutes early for this and I was 10 minutes late, just finding the email, getting in, getting the passwords to work. It's usually annoying and it was occasionally prohibitive. I know that's sort of the point, I mean, it's better than needing to drive somewhere or something like that but at the same time it's just like…..That's super disappointing so the technology was sort of a drag.”*

**Fig. 2:**
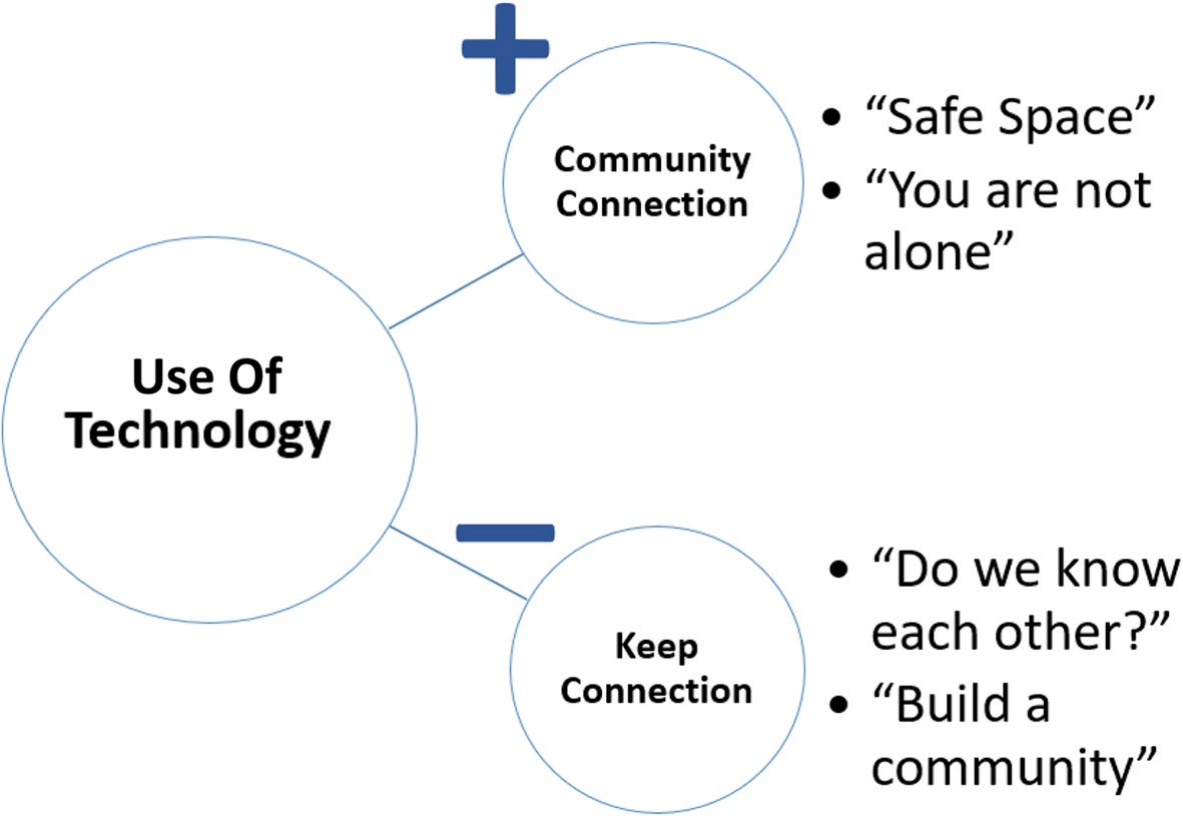
Use of technology impact on other categories

Use of technology also had positive and negative impacts on other categories. Figure 2 shows how positive/negative uses of technology experiences effect other categories: community connection and keeping a connection.

Use of technology may have a positive effect on community connection through convenience of technology. A web-based video chat room provided privacy, which led to participants feeling that they had a “safe place” to talk with other women who were “going through a similar thing.” Privacy offered participants a platform where they could be open to express their feelings. This led to participants feeling that they “were not alone.” Although technology allowed participants to have face-to-face interaction and fostered communication during the group sessions, one of the drawbacks was that participants found this secluded video chat room environment somewhat prohibiting in building personal one-on-one relationships. At the conclusion of the sessions, women often turned off their video chat immediately, limiting opportunities to connect with each other pre- and post-session, a different experience from an in-person group. Participants expressed their desire to keep connected with each other between sessions or on their own time. Participants suggested that they would like to keep connected to each other and “build a community” of support after sessions had ended.

## Discussion

The idea of connection was the centralized theme in both the primary and secondary analyses, tying the core themes of community with women experiencing PD, normalization of depression, and reduced stigma of depression together. Feedback provided by the participants elucidated whether the use of technology helped to maintain the sense of community and connection or detracted from the program.

Feedback from participants was overwhelmingly positive, with many women expressing hope that the program would continue in the future. All of the participants across the three groups described the VC UPLIFT as helpful in reducing feelings of isolation and increasing a sense of having a common experience. This is important because isolation is a common experience among peripartum women with PD in both urban and rural settings. Urban pregnant women with depression report feelings of intense isolation and loneliness [32]. Similarly, rural pregnant women report that transportation and isolation/loneliness are predominant stressors for them [27].

Women described the intervention using videoconference system as convenient, as they did not have to find childcare or transportation, despite minor technical difficulties at times. This provided women with the opportunity to participate in a group intervention in which they would not otherwise have had the ability or motivation to participate. This indicates that interventions delivered via telehealth may address gaps in access to care for such women. Additionally, telehealth may be the only option available where there is limited access to mental health providers. In at least one study, nearly 36% of rural pregnant women met criteria for depression, yet approximately 92% were not receiving any type of mental health services [33]. This is more than double the approximate 15% of pregnant women overall who experience depressive symptoms, where 50% do not receive mental health services. These statistics are in alignment with common barriers to mental health services, such as isolation, transportation, and shortage of mental health providers reported in rural communities [13, 15, 16]. Based on the results of our qualitative study, a group telehealth intervention is a promising platform for addressing isolation and reducing barriers to accessing mental health services for childbearing women in both urban and rural communities.

In contrast to the isolation often felt among depressed pregnant women, the VCI provided participants with a sense of universality, normalization of their experiences, and reduced stigma of mental health issues. As technology-mediated support groups increase in number, it is necessary to explore whether therapeutic factors found in face-to-face group settings may or may not be present in digital forums and/or how these might be better integrated into telehealth. For example, universality is known as a common curative factor of groups and is highly correlated with participants’ perceived helpfulness of a group [34]. Universality appears to be present in both online and in-person group sessions. However, technology may not be an ideal platform for establishing deeper connections with others, in comparison to in-person groups. Fostering interactions between and after VCI sessions might mitigate this perception.

Opportunities for deeper connections with peers outside the group setting are more limited and future interventions should explore this telehealth limitation to enhance connection within group care. This study provides data supporting universality through a telehealth intervention. This matches other research findings that therapeutic factors, such as universality and shared common experiences among group members, are present in telehealth support groups [35].

Based on participant suggestions, future programs could include a structure or means for patients to interact outside of weekly group meetings. Participants recommended platforms to support this such as a private social media group, email lists, or instant messaging/group texting. Participants also recommended extending sessions well into the postpartum period (beyond 8 weeks) as a means to provide continued support to women transitioning into motherhood and to protect against postpartum depression. Some participants also recommended in-person sessions or meet-ups beyond the VCI, although many expressed that they would likely not attend. However, some viewed in-person sessions as a means to establish greater trust among group attendees and offer peer support, should the need arise. Also, the likely geographic distance between participants would not be conducive to in-person interactions.

The greatest drawbacks to the VCI were related to the occurrences of VC instability and difficulty communicating when technological problems arose. The most common technical difficulties for some women were directly related to internet resources, such as weak wireless connections and insufficient bandwidth for video conferencing. These challenges may be more prevalent in rural areas. Prior to attending the VCI, each participant met with a study team member to test the individual’s technology, practice connecting and using the platform, and to troubleshoot any difficulties. This process significantly reduced the occurrence of technical problems, but did not eliminate them, particularly for those participants with inadequate internet connections. Provision of direct technology support and timely troubleshooting of technical issues can lead to more positive experiences.

Other approaches to offset difficulties could include arranging for adequate internet bandwidth in women’s homes, or collaborating with local community facilities, such as libraries or public health clinics to provide adequate internet connections and private settings for women to participate. Ideally, rapid access to a technical support person who could assist with troubleshooting is important, as well as encouragement to use telephone connection during the session, as a quick alternative to videoconferencing. Furthermore, it is important to explore how telehealth environments can better support personal connections amongst individuals similar to in-person interactions. Addressing the suggestions that women made during the focus groups should be integrated in order to maximize the effectiveness of this and other telehealth intervention programs.

### Limitations

The study had a limited number of participants and only 17 out of 47 (36%) participated in a focus group. This could potentially bias results, as those who participated in a focus group may be different than those who did not; those who were less satisfied may have been less likely to participate, and conversely, those who were more satisfied may have been more likely. To address this potential bias and to effectively identify barriers and solutions to successful telehealth group mental health programs, future research should focus on individuals who decline or discontinue participation in telehealth programs, or those who do not reap benefits.

The inclusion of a limited number of Hispanic women (reflecting Utah’s population at large) and other women of color in the study limits the generalizability of findings to those that participated in the study (Table 2). The two women of color in the group vocalized the need for prenatal providers to initiate conversations about mental health, and about the stigma of mental health. However, because there were only two participants of color, it is unclear if this emphasis was related to ethnic identity. Follow-up is needed to understand varying racial/ethnic experiences.

Finally, as the women did not engage with one another outside of the VCI sessions, it is unknown whether creating intentional connections outside of the group may enhance the positive outcomes of the intervention; further study is needed.

## Conclusion

This study provides evidence that women overall have a positive experience when engaging in a PD oriented group telehealth intervention. Utilizing a VC to deliver a MBCBT program to women who are at risk for or experiencing symptoms of PD/anxiety has the potential to reduce barriers to accessing care for women regardless of their geographical location, while reducing costs. The rapid expansion of telehealth during the COVID-19 pandemic has exposed a larger percentage of the population to telehealth. Additionally, providers and health systems have established telehealth platforms for service delivery and have gained experience using these platforms and troubleshooting connection challenges. These factors greatly increase the potential for more widespread utilization of telehealth for the provision of mental health care in the future.

To maximize positive effects of telehealth intervention programs, reducing barriers around the use of technology is critical. Troubleshooting as soon as technical difficulties are experienced may optimize benefits of using technology. Furthermore, it is important to explore how to foster personal connections amongst individuals who are participating in telehealth group programs, similar to the in-person connections. The perspectives shared by women in these focus groups can be leveraged to improve group telehealth designed to address PD, and inform future research that evaluates the format and delivery of effective telehealth-based group mental health services.

## Data Availability

The datasets generated and/or analysed during the current study are not publicly available due to this being a qualitative study with transcript data that could possibly be identifiable but deididentfied transcripts could be available from the corresponding author on reasonable request.

## Abbreviations

EPDS: Edinburgh Postnatal Depression Scale
MBCBT: Mindfulness-Based Practices and Cognitive Behavioral Therapy
UPLIFT: Using Practice and Learning to Increase Favorable Thoughts
VCI: Videoconference Intervention
